# Effects of Resveratrol on Anxiety-Like Behavior, Neuronal Damage, and Apoptotic Signaling in Female Rats Exposed to Methylparaben

**DOI:** 10.7150/ijms.138134

**Published:** 2026-09-03

**Authors:** Entesar AlShammari, Promy Virk, Dalal Alkhelb, Fawaz Alasmari, Wasayf Alotibi, Nouf Alsultan, Gadah Albasher

**Affiliations:** 1 Department of Zoology, College of Science, King Saud University, Riyadh, Saudi Arabia.; 2 Department of Pharmacology and Toxicology, College of Pharmacy, King Saud University, Riyadh, Saudi Arabia.; 3 Faculty of Medicine and Health Sciences, Medical School, University of Nottingham, Nottingham NG7 2QL, UK.

**Keywords:** resveratrol, methylparaben, anxiety, neuroinflammation, neurobiology

## Abstract

Methylparaben (MEP), a widespread preservative, raises neurotoxicity concerns through oxidative stress and inflammation. Resveratrol (RES) is a natural polyphenol with antioxidant and anti-inflammatory properties. This study investigated whether RES protects against MEP-induced neurotoxicity in female rats. Female Wistar rats were divided into five groups (n=10/group): control, vehicle (5% DMSO), MEP alone (200 mg/kg, s.c., 15 days), RES alone (20 mg/kg, i.p., 15 days), and MEP+RES co-treatment. We evaluated anxiety-like behavior (elevated plus maze), brain histopathology (cortex/hippocampus), oxidative stress markers (GSH, SOD), stress-related gene expression (*Nrf2, ASK1, PP-1cs, PP-2Acs* by qPCR*)*, inflammatory proteins (TNF-α, IL-6 by immunohistochemistry), and apoptotic markers (BAX, BCL-2). MEP exposure significantly increased anxiety-like behavior, caused structural damage in cortex and hippocampus, depleted GSH and SOD, upregulated *Nrf2, ASK1, PP-1cs*, and *PP-2Acs*, elevated TNF-α, IL-6, and pro-apoptotic BAX, while reducing BCL-2. RES alone caused no adverse effects. Remarkably, RES co-administration significantly attenuated and partially restored MEP-induced alterations, restoring behavior, tissue architecture, antioxidant defenses, gene expression, and immune balance. RES effectively attenuates MEP-induced neurotoxicity through antioxidant, anti-inflammatory, and anti-apoptotic mechanisms, positioning it as a promising neuroprotective agent against environmental toxicants.

## 1. Introduction

Methylparaben (MEP), an ester of para-hydroxybenzoic acid, is frequently used as a preservative in personal care products, pharmaceuticals, and food products 1. Despite its widespread use, recent studies have raised concerns about its potential behavioral and genotoxic effects 2,3. In several experimental studies, chronic co-exposure of zebrafish to parabens interfered with learning, memory, anxiety, fear, aggression, and shoaling behaviors 4,5. Additionally, parabens have been shown to cross the blood-brain barrier, and its neurotoxicity has been linked to oxidative stress, inflammation, and neuronal damage 4,6,7. More critically, studies in children have shown that the presence of parabens in children's urine, including MEP, is linked to reduced performance in girls compared to boys, highlighting a sex difference in the neurodevelopmental effects of MEP 8.

While data on the neurotoxic effects of MEP exist from zebrafish studies, further examination in more complex laboratory animal models is warranted to determine whether MEP induces cognitive decline or anxiety-like behaviors. Findings from such studies are crucial for regulatory agencies to reassess the safety of MEP and to consider the potential long-term risks associated with chronic exposure. In addition, identifying effective preventative and treatment strategies to reduce the risk of MEP-induced behavioral and neuronal toxicity is needed to address this concern 5.

Resveratrol (RES), a natural polyphenolic compound present in grapes, red wine, and various berries, has demonstrated neuroprotective properties 9,10. RES exerts its effects through multiple pathways, including the enhancement of antioxidant defense mechanisms, the suppression of pro-inflammatory mediators, and the modulation of cellular signaling pathways involved in stress response 11,12. Given these multifaceted protective mechanisms, RES holds promise as a therapeutic agent for alleviating the detrimental effects of environmental and pharmaceutical toxicants.

This study investigates the neuroprotective effects of RES in counteracting MEP-induced neurotoxicity in female rats. Behavioral tests, gene expression analyses of oxidative stress markers, and histopathological assessments of the cortex and hippocampus were performed to clarify the protective effects of RES and its potential mechanisms of action.

## 2. Materials and Methods

### 2.1 Materials

Methylparaben (MEP; CAS No. 99-76-3; purity ≥ 99.5%) was obtained from MYOC Chemical Co. (New Delhi, India). Resveratrol (RES; purity ≥ 99.5%) was purchased from Bulk Supplements (Henderson, NV, USA). Dimethyl sulfoxide (DMSO) was sourced from Sigma-Aldrich (St. Louis, MO, USA).

MEP and RES were each dissolved in DMSO and diluted with sterile phosphate-buffered saline (PBS) to achieve a final DMSO concentration of 5% (v/v). Both solutions were prepared fresh daily. MEP was administered subcutaneously at a dose of 200 mg/kg body weight in a volume of 0.5 mL per animal, while RES was administered intraperitoneally at a dose of 20 mg/kg body weight in a volume of 0.5 mL per animal and protected from light due to its photosensitivity.

All solutions were stored at 4 °C when not in use and discarded after 24 hours.

### 2.2 Experimental Animals

Female Wistar rats, aged 16 weeks and weighing 180-200 g at the beginning of the experiment, were obtained from the Animal House, Department of Zoology, College of Science, King Saud University, Riyadh, Saudi Arabia. The animals were maintained under standard laboratory conditions, including a 12-h light/dark cycle, a controlled ambient temperature of 25 ± 1 °C, and relative humidity of 50 ± 10%. All rats had free access to standard commercial rodent chow and tap water ad libitum. Body weights were recorded at the beginning and at the end of the experimental period, and the animals were monitored daily for general health status and signs of toxicity throughout the study.

Female rats were selected because MEP is commonly used in cosmetic and personal-care products that are more frequently used by women; thus, female animals provide a biologically relevant model for potentially higher exposure. MEP is also classified as an endocrine-disrupting compound, making female animals particularly relevant to investigate possible effects on female physiology. Additionally, female rats generally exhibit lower levels of aggression and social stress than males, which can reduce experimental variability and improve consistency of outcomes 1,8.

All experimental procedures were approved by the Institutional Animal Ethics Committee of King Saud University (Approval No. KSU-SE-24-32) and were conducted in accordance with the National Institutes of Health (NIH) Guide for the Care and Use of Laboratory Animals (8th edition, National Research Council, 2011) and all applicable institutional guidelines for the care and use of laboratory animals.

### 2.3 Experimental Design

Female Wistar rats were randomly allocated into five experimental groups (n = 10 per group) with different doses of treatments 13- 15 as follows:

**Group 1 (Negative Control):** served as the baseline control for normal physiological, behavioral, and histological parameters.

**Group 2 (Vehicle Control - DMSO):** received daily intraperitoneal (i.p.) injections of 5% dimethyl sulfoxide (DMSO) in sterile phosphate-buffered saline (PBS) at a volume of 0.5 mL per animal for 15 consecutive days. This group controlled for any potential effects of the solvents used to dissolve MEP and RES.

**Group 3 (Methylparaben - MEP):** received daily subcutaneous (s.c.) injections of MEP at a dose of 200 mg/kg body weight in a volume of 0.5 mL for 15 consecutive days. Injections were administered alternately on both sides of the inguinal region 15.

**Group 4 (Resveratrol - RES):** received daily intraperitoneal (i.p.) injections of RES at a dose of 20 mg/kg body weight in a volume of 0.5 mL for 15 consecutive days 13.

**Group 5 (MEP + RES):** received daily concurrent injections of both MEP (200 mg/kg, s.c., inguinal region) and RES (20 mg/kg, i.p.) for 15 consecutive days. The two injections were administered at separate sites on each animal to avoid any potential local interaction 14.

The sample size (n = 10 animals per group) was selected based on previous studies investigating paraben-induced neurotoxicity and the neuroprotective effects of RES using comparable behavioral, biochemical, and histopathological endpoints 16-17 A formal a priori power analysis was not performed; however, the selected sample size was considered sufficient to detect biologically relevant differences between experimental groups while adhering to the principles of reduction in animal experimentation.

The selected MEP dose (200 mg/kg, s.c., for 15 days) was used intentionally as an acute/high-dose proof-of-concept to produce robust oxidative, inflammatory, and apoptotic insults for testing resveratrol's therapeutic capacity. For clarity, with an average adult female Wistar rat weight of ~200 g, this corresponds to ≈40 mg MEP per animal per day. Using standard allometric scaling (Km_rat = 6; Km_human = 37) the approximate human-equivalent dose (HED) is ≈200 × (6/37) ≈ 32.4 mg/kg, which for a 70-kg adult equates to ~2.27 g/day. These values are therefore several orders of magnitude higher than typical human environmental exposures to MEP (generally in the ng-µg/kg range). Accordingly, our model is intended to demonstrate proof-of-concept efficacy of RES under pronounced acute toxic stress rather than to directly represent routine chronic human exposure 15.

### 2.4 Behavioral analysis

Behavioral assessment of anxiety-like behavior was conducted using the Elevated Plus Maze (EPM). Each rat was tested individually for 5 minutes. Behavioral parameters were recorded through direct visual observation, and the number of entries into the open and closed arms, as well as the time spent in each arm, were counted manually. The test was conducted under dim red light to minimize stress and avoid influencing the animals' natural behavior 18.

### 2.5 Evaluation of antioxidant defense markers: glutathione (GSH) and superoxide dismutase (SOD)

The antioxidant markers glutathione (GSH) and superoxide dismutase (SOD) were assessed using commercially available assay kits according to the manufacturers' instructions. GSH levels were determined using a competitive ELISA kit (ELK Biotechnology, Wuhan, China; Cat. No. ELK10914), while SOD activity was measured using a colorimetric assay kit (Sigma-Aldrich, Merck, Germany; Cat. No. CS0009). Tissue samples were homogenized in phosphate-buffered saline (PBS) and centrifuged to obtain the supernatant for analysis. All assays were performed in 96-well microplates, and absorbance was measured at 450 nm using a microplate reader. The concentrations and enzyme activities were calculated based on the respective standard curves. Each experimental group consisted of ten animals (n = 10). All biochemical assays were performed in duplicate or triplicate according to assay requirements, and the mean values were used for statistical analysis.

### 2.6 Histopathological processing of brain tissues

Brain tissues were fixed in 10% formalin, dehydrated, embedded in paraffin, sectioned, and stained with hematoxylin and eosin. Sections were examined and imaged using a light microscope (Nikon, Japan) 19.

### 2.7 Immunohistochemistry analysis

Immunohistochemical staining was performed using reagents from Santa Cruz Biotechnology according to the manufacturer's instructions. Tissue samples were fixed, embedded in paraffin, and sectioned into 8 µm-thick sections using a microtome. The sections were deparaffinized, rehydrated, and subjected to antigen retrieval in EDTA (pH 9) to enhance antigen accessibility and membrane permeabilization. Non-specific binding sites were then blocked. Sections were then incubated with primary antibodies against IL-6, TNF-α, BAX, and BCL-2 (My BioSource, USA), (all anti-rabbit) overnight at 4 °C, followed by incubation with the appropriate secondary antibody for 1 hour and then with avidin-biotin for 1 hour. Immunoreactivity was visualized using 3,3′-diaminobenzidine (DAB) as a chromogenic substrate. Finally, the sections were analyzed using Fiji Software to obtain distribution area (%) and optical density, calculated as log (Max/Mean) × 100, where Max represents the maximum intensity and Mean represents the mean intensity. Sections were then mounted and examined under a light microscope.

### 2.8 Quantitative analysis of gene expression by RT-qPCR

Quantitative polymerase chain reaction *(qPCR)* was conducted to measure the expression of oxidative stress and inflammation-related genes, including mRNA expression of nuclear factor erythroid-derived 2 (*Nrf2*) (Table 1). In addition, expression of apoptosis signal-regulating kinase 1 (*ASK1*) was assessed. The mRNAs for the protein phosphatases PP-1 catalytic subunit *(PP-1cs)* and *PP-2A* catalytic subunit *(PP-2Acs)* were also assessed. RT-qPCR was conducted using a SYBR Green system.

RT-qPCR was performed using a real-time PCR system (ViiA™ 7, Applied Biosystems, Thermo Fisher Scientific, USA) with a SYBR Green-based detection system (Bio-Rad Laboratories, USA). Primers were synthesized by Macrogen Inc. and supplied in lyophilized (freeze-dried) powder form. Primers were reconstituted in nuclease-free water and stored at -20 °C. The qPCR protocol from Bio-Rad Laboratories, Inc. using SYBR Green was strictly followed. All procedures were conducted according to the company's standardized guidelines to ensure accuracy, reproducibility, and compliance with quality standards. The expression level of the *GAPDH* transcript was measured as a housekeeping gene. The analysis and calculation of gene expression was performed according to a previous study 20.

PCR amplification was carried out under the following conditions:

Initial denaturation at 95 °C for 5 min, followed by 40 cycles of denaturation at 95 °C for 15 s, annealing at 60 °C for 30 s, and extension at 72 °C for 30 s. Relative gene expression levels were calculated using the 2-ΔΔCt method for relative quantification as previously described 20-22.

### 2.9 Statistical analysis

Data were analyzed using GraphPad Prism version 10.0 (GraphPad Software, Boston, MA, USA). Differences between groups were analyzed using a one-way analysis of variance (ANOVA). When significant differences were found, they were followed by Tukey's HSD post-hoc test. A p-value of less than 0.05 was considered statistically significant. Data are presented as mean ± standard error of the mean (SEM).

## 3. Results

### 3.1 Assessment of anxiety-like behavior using the elevated plus maze

In the maze test, no significant differences were observed among the control, DMSO, and RES-treated groups in either closed-arm entry frequency or time spent in the closed arms (Fig. 1). However, the MEP-treated group showed a significant increase in both parameters compared with the control group, indicating enhanced anxiety-like behavior. Co-administration of RES with MEP significantly reversed these alterations, as evidenced by a reduction in closed-arm entries and time spent in the closed arms compared with the MEP-treated group.

### 3.2 Evaluation of antioxidant defense markers: glutathione (GSH) and superoxide dismutase (SOD)

Antioxidant levels of GSH and SOD showed no significant changes in the DMSO or RES groups relative to controls (*p* > 0.05; Fig. 2). In contrast, MEP-treated animals exhibited a significant decrease in both GSH and SOD compared to the control group (*p* ≤ 0.05; Fig. 2). Co-treatment with RES (MEP + RES) significantly restored these antioxidant levels compared to the MEP group alone (*p* ≤ 0.05; Fig. 2).

### 3.3 Real-time PCR quantification of gene expression

#### 3.3.1 qRT-PCR analysis of Nrf2

RT-qPCR analysis of Nrf2 expression in rat brain tissue, normalized to GAPDH as the endogenous control, revealed that MEP treatment significantly upregulated Nrf2 compared to the control group. This upregulation indicates increased oxidative stress following MEP administration. In contrast, co-administration of RES with MEP significantly downregulated Nrf2 expression compared to the MEP group alone, reflecting a reduction in oxidative stress due to RES treatment (Fig. 3).

#### 3.3.2 qRT-PCR Analysis of *ASK1*

qRT-PCR analysis revealed that MEP administration significantly increased *ASK1* expression in brain tissue compared to the control group, reflecting activation of oxidative and pro-apoptotic pathways through *ASK1* induction. In contrast, co-administration of RES with MEP significantly downregulated *ASK1* expression compared to the MEP group alone, demonstrating that RES suppresses *ASK1* and inhibits these pro-apoptotic pathways (Fig. 4).

#### 3.3.3 qRT-PCR Analysis of PP-1cs

qRT-PCR analysis revealed that MEP treatment significantly upregulated *PP-1cs* mRNA expression compared to the control group, reflecting increased activation of stress-response pathways. Furthermore, treatment with RES in combination with MEP resulted in marked downregulation of *PP-1cs* compared to the MEP group alone, indicating a decrease in stress responses (Fig. 5).

#### 3.3.4 qRT-PCR Analysis of *PP-2Acs*

qRT-PCR analysis revealed that MEP treatment significantly upregulated PP-2Acs mRNA expression compared to the control group, indicating disruptions in lipid metabolism, cellular stress responses, and inflammatory signaling (Fig. 6).

### 3.4 Histopathological evaluation of brain architecture

#### 3.4.1 Histopathological assessment of cerebral cortex

The cerebral cortex of control animals appeared normal, showing abundant pyramidal and granular neurons along with glial cells in the neuropil (Fig. 7A). Animals treated with DMSO or RES exhibited the same normal cortical structure (Fig.7B, C). In contrast, MEP-treated animals showed severe cortical alterations, including widespread hemorrhage, inflammatory cell infiltration, neuronal enlargement, and increased glial cells (Fig. 7D). Notably, co-treatment with RES (MEP + RES) markedly improved cortical architecture, with no hemorrhage observed aside from mild blood vessel dilation and a small number of inflammatory cells (Fig. 7E).

#### 3.4.2 Histopathological investigation of hippocampus

The control hippocampus exhibited normal architecture, with a distinct granular cell layer situated between the molecular and polymorphic layers (Fig. 8A). Animals treated with DMSO or RES showed similarly normal hippocampal structure, with no pathological alterations (Fig. 8B, C). In contrast, MEP-treated animals displayed marked neuronal distortion and enlargement in most hippocampal neurons (Fig. 8D). Notably, co-treatment with RES (MEP + RES) reduced this damage, with only a small number of distorted neurons remaining compared to the MEP group (Fig. 8E).

### 3.5 Target-specific immunohistochemical determination of immune expressions

#### 3.5.1 Expression of inflammation mediators TNF-α and IL6

Inflammatory mediator expression (TNF-α and IL-6) was weak in the control group (Fig. 9A). Similarly, animals treated with DMSO or RES alone showed low levels of these inflammatory markers (Fig. 9B, C), reflected in low distribution area and optical density percentages (Fig. 10A, B). In contrast, MEP-treated animals exhibited intense, dark immunostaining in the cerebral cortex (Fig. 9D), with markedly higher distribution area and optical density percentages compared to controls (Fig. 10A, B). Notably, co-treatment with RES (MEP + RES) significantly reduced these inflammatory expressions, as evidenced by decreased distribution area (Fig. 9E) and optical density percentages (Fig. 10A, B) relative to the MEP group.

Similarly, IL-6 expression followed the same pattern: weak immunostaining in control, DMSO, and RES groups (Fig. 11A,C), intense staining in the MEP group (Fig. 11D), and reduced staining in the MEP + RES group (Fig. 11E). Quantitative analysis confirmed significantly higher distribution area and optical density for IL-6 in the MEP group compared to controls, with significant reduction following RES co-administration (Fig. 12A, B).

### 3.6 Oxidative stress-related mediator expression BAX and BCL_2_

The control cerebral cortex exhibited weak BAX expression and strong BCL-2 expression (Fig. 13A; Fig. 15A). Animals treated with DMSO or RES alone showed similar patterns, with low BAX levels and high BCL-2 levels (Fig. 13B, C; Fig. 15B, C). Quantitative analysis revealed that the control group had a BAX distribution area of 2% and optical density of 1%, while the DMSO and RES groups showed comparably low values (DMSO: 3% distribution, 2% optical density; RES: 4% distribution, 3% optical density), with no significant differences between these groups (ns) (Fig. 14A, B).

In contrast, MEP-treated animals displayed increased BAX expression and reduced BCL-2 expression compared to controls, indicating elevated BAX and decreased BCL-2 levels (Fig. 13D; Fig. 15D). Quantitatively, the MEP group showed a marked elevation in BAX expression, with a distribution area of 35% and an optical density of 30% (**p < 0.0001 vs. control) (Fig. 14A, B). For BCL-2, the MEP group exhibited a significant reduction in distribution area and optical density compared to the control group (*p < 0.001; ****p < 0.0001) (Fig. 16A, B).

Notably, co-treatment with RES (MEP + RES) significantly reduced BAX expression and increased BCL-2 expression compared to the MEP group alone, resulting in decreased BAX levels and elevated BCL-2 levels (Fig. 13E; Fig. 15E). The MEP + RES group showed a BAX distribution area of 22% and optical density of 18% (****p < 0.0001 vs. MEP), indicating a protective anti-apoptotic effect of RES (Fig. 14A, B). For BCL-2, co-treatment with RES partially restored expression relative to the MEP group alone (*p < 0.05; **p < 0.01; ***p < 0.001), further supporting the anti-apoptotic protective effect of RES (Fig. 16A, B).

## 4. Discussion

In this study, we investigated whether MEP induces neurotoxicity in female rats and, if so, whether RES confers protection. Parabens such as MEP are among the most widely used preservatives in cosmetics, lotions, pharmaceuticals, and processed foods. However, growing evidence suggests that prolonged exposure to these compounds may adversely affect the nervous system 1,3. In fact, both experimental and environmental studies have linked MEP exposure to disruptions in neuronal integrity and neurobehavioral toxicity, primarily through mechanisms involving oxidative stress, neuroinflammation, and the activation of apoptotic pathways.

Our behavioral assessment using the Elevated Plus Maze revealed that rats exposed to MEP exhibited a significant increase in both the frequency of closed-arm entries and the time spent in the closed arms compared to control animals. In behavioral neuroscience, the Elevated Plus Maze and Light-Dark Box are well-recognized indicators of heightened anxiety 23- 24. The most plausible explanation is that MEP triggers neurochemical imbalances and neuronal dysfunction driven largely by oxidative stress and neuroinflammation. This interpretation is supported by earlier studies showing that paraben exposure can alter neurobehavioral responses and provoke anxiety-like behavior by disrupting neurotransmitter systems and stress hormone regulation 5. Importantly, animals that received RES alongside MEP showed a significant reduction in these anxiety-related behaviors, suggesting that RES exerted genuine anxiolytic-like effects, likely by protecting the neuronal circuits involved in emotional regulation. These findings align with previous work demonstrating that RES improves behavioral performance and cognitive function in various models of neurological impairment 25.

To determine whether RES reduced MEP-induced oxidative damage at the biochemical level, we measured two key antioxidant defenders: glutathione (GSH) and superoxide dismutase (SOD). MEP exposure significantly lowered both GSH levels and SOD activity in brain tissue. This is critical because GSH and SOD serve as the body's primary defense against reactive oxygen species, maintaining cellular redox balance. Their depletion allows oxidative stress to prevail, as observed in our MEP-treated animals. These findings are consistent with earlier reports that paraben exposure disrupts antioxidant enzyme systems and promotes oxidative damage in experimental models 19 Oxidative stress is a major driver of neuronal injury, fueling lipid peroxidation, impairing mitochondrial function, and initiating inflammatory cascades 26 Notably, co-administration of RES with MEP significantly restored both GSH and SOD levels, demonstrating that resveratrol's well-established ability to scavenge free radicals and boost antioxidant defenses 16,27,28 extends to the brain against MEP-induced injury.

Neuronal examination of brain sections from MEP-treated rats revealed increased oxidative stress and altered proteome profiles in the brains. Observed alterations included altered neurotransmission, elevated LPS endotoxin infiltration into male brains leading to increased synthesis of pro-inflammatory cytokines 29. These findings indicate ongoing neuronal degeneration and a neuroinflammatory response triggered by MEP, consistent with similar histopathological changes reported after paraben exposure 26. Remarkably, animals co-treated with RES showed marked improvement, with near-normal cortical architecture and substantially reduced inflammatory infiltration. Together with our biochemical data, these histological findings support that RES preserves neuronal integrity against MEP-induced neurotoxicity.

To explore molecular mechanisms, we used immunohistochemistry to examine the inflammatory mediators tumor necrosis factor alpha (TNF-α) and interleukin-6 (IL-6). In the cerebral cortex of MEP-treated animals, both cytokines were significantly overexpressed. Elevated TNF-α and IL-6 are not merely markers but active drivers of neuroinflammation that exacerbate neuronal injury and promote neurodegeneration. However, studies reported that RES showed ability to restore neurobiological proteins and attenuate inflammatory responses in experimental models 11,30.

In addition to inflammation, MEP promoted apoptosis (programmed cell death). This was evident from the altered balance between the pro-apoptotic protein BAX and the anti-apoptotic protein BCL-2. MEP-treated animals exhibited increased BAX expression and decreased BCL-2 levels. This shift is likely driven by oxidative stress: excessive reactive oxygen species attack mitochondria, disrupting membrane integrity, promoting lipid peroxidation, and activating apoptotic cascades. The resulting increase in mitochondrial membrane permeability favors pro-apoptotic mediators like BAX while suppressing anti-apoptotic defenders like BCL-2, pushing neurons toward death. In RES-co-treated animals, BAX expression decreased and BCL-2 levels recovered. We attribute this to RES's potent antioxidant activity, which scavenges free radicals, boosts endogenous antioxidant defenses, and preserves mitochondrial function, thereby interrupting MEP-triggered apoptotic signaling.

The use of a short-term, high-dose MEP paradigm was deliberate to generate clear mechanistic insults (oxidative stress, neuroinflammation, apoptosis) and to test whether RES can ameliorate those outcomes. Acute high-dose paradigms are appropriate for demonstrating therapeutic potential but differ fundamentally from chronic low-level human exposures; dose-dependent and hormetic responses have been reported, and the protective mechanisms of RES have been described previously 9, 28. Therefore, while our findings provide strong proof-of-principle that RES mitigates pronounced toxic injury, they should be interpreted within this experimental context and motivate further studies assessing RES efficacy under exposure routes, doses, and durations more representative of human chronic exposure **10,16.**

Using allometric scaling, the animal dose (200 mg/kg) corresponds roughly to an HED of ~32.4 mg/kg, which remains far above environmental human exposure levels (ng-μg/kg). Therefore, our findings demonstrate RES efficacy in a high-dose acute toxicity context and do not directly indicate risk from typical chronic human exposures.

The exclusive use of female animals was intentional to reflect a population potentially at higher exposure and to probe sex-relevant endocrine effects 31. However, results may not fully generalize to males and future studies should include both sexes 8.

At the genetic level, RT-qPCR revealed that MEP exposure significantly upregulated four key genes involved in oxidative stress and stress-response signaling: Nrf2, ASK1, PP-1cs, and PP-2Acs. Nrf2 is a master regulator of antioxidant defense, and its upregulation most likely reflects an adaptive cellular response to increased oxidative stress. ASK1 sits at the crossroads of stress signaling and can promote apoptotic cell death, whereas PP-1cs and PP-2Acs participate in cellular stress responses and inflammatory signaling pathways. The concurrent upregulation of these genes suggests that MEP exposure elicited a robust cellular stress response characterized by increased oxidative and inflammatory challenges. In particular, the elevated Nrf2 expression most likely represents a compensatory antioxidant response aimed at counteracting excessive oxidative stress rather than a direct marker of oxidative damage itself 32.

RES co-administration significantly reduced the expression of all four genes, suggesting an overall attenuation of cellular stress and restoration of redox homeostasis. The reduction in Nrf2 expression observed following RES treatment may reflect a decrease in the oxidative stimulus that triggered compensatory Nrf2 induction rather than direct inhibition of the Nrf2 signaling pathway by RES, indicating that RES alleviated oxidative burden and helped restore cellular homeostasis. These findings are consistent with previous studies demonstrating that RES attenuates oxidative stress and exerts neuroprotective effects against toxic insults 17. However, because only Nrf2 mRNA expression was assessed in the present study, without evaluation of Nrf2 protein levels or downstream target genes such as HO-1 and NQO1, definitive conclusions regarding activation or inhibition of the Nrf2 pathway cannot be drawn, and the present findings should therefore be interpreted with caution 16. Taken together, our findings indicate that MEP induces neurotoxicity through multiple interconnected mechanisms: oxidative stress, neuroinflammation, and activation of apoptotic pathways. These pathological processes ultimately lead to structural neuronal damage and the observed behavioral alterations, and we hypothesize that RES produces protective effects 33.

Before concluding, we address an interesting point of comparison with the existing literature. Kopalli and colleagues 34 reported that MEP protected against neurotoxicity in a mouse model of Parkinson's disease—essentially the opposite of our findings. At first glance, this appears contradictory. However, the explanation likely lies in dosing. That study used very low doses of MEP (1, 10, or 50 μg/kg, administered intraperitoneally), whereas we used a much higher dose (200 mg/kg, dissolved in DMSO). In toxicology, dose determines the effect. Low doses of certain compounds can sometimes trigger beneficial hormetic responses—mild stress that activates protective pathways—while high doses overwhelm those same systems and cause damage. Rather than viewing our results as conflicting, we consider them complementary, demonstrating that MEP's effects on the nervous system are highly dose-dependent.

Protein phosphatase-1 catalytic subunit (PP-1cs) and protein phosphatase-2A catalytic subunit (PP-2Acs) are major serine/threonine phosphatases that regulate neuronal signaling, synaptic plasticity, apoptosis, and neuroinflammatory responses 35,36 PP-1 is critically involved in the modulation of learning, memory, and emotional behavior through the regulation of synaptic proteins and neurotransmitter receptor phosphorylation. Dysregulation of PP-1 signaling has been associated with impaired synaptic function, increased neuronal vulnerability, and anxiety-like behaviors 35 Similarly, PP-2A serves as a key regulator of neuronal survival by controlling the phosphorylation status of several proteins involved in oxidative stress responses, mitochondrial integrity, and apoptotic pathways 36 Reduced PP-2A activity has been linked to excessive neuroinflammation, abnormal protein phosphorylation, and activation of pro-apoptotic mediators, including p53 and Bax 36,37.

The significant alterations in PP-1cs and PP-2Acs expression observed in the present study suggest that MEP-induced neurotoxicity involves disruption of phosphatase-mediated signaling pathways. The increased expression of these phosphatases was accompanied by anxiety-like behavior, elevated inflammatory mediators (TNF-α and IL-6), and enhanced apoptotic responses in the cortex and hippocampus, indicating a close association between phosphatase dysregulation and neuronal injury. The restoration of PP-1cs and PP-2Acs expression by RES suggests that modulation of phosphorylation-dependent signaling pathways may represent an additional mechanism underlying its neuroprotective effecs 35-37.

It should be noted that, although the present findings demonstrate a significant association between reduced oxidative stress, modulation of molecular markers, and improved behavioral outcomes following RES treatment, direct causal relationships between specific signaling pathways, particularly Nrf2 activation, and the observed neurobehavioral improvements cannot be definitively established within the current experimental design 36 Since pathway-specific inhibitors or genetic approaches were not employed, the mechanistic interpretations presented herein should be considered associative rather than causal. Future studies using targeted pharmacological inhibitors or genetic models are warranted to confirm the precise molecular mechanisms underlying the neuroprotective effects of RES.

## Conclusion

This study provides clear experimental evidence that RES protects against MEP-induced neurotoxicity in female rats. MEP triggers oxidative stress, fuels neuroinflammation, and pushes neurons toward apoptosis, leading to structural brain damage and behavioral changes. However, dose matters: low doses of MEP have been reported to have protective effects in some contexts 34,while the higher dose used here produced clear toxicity. These findings are not in conflict but rather highlight the importance of dose in toxicology. What remains unambiguous in our study is the protective power of RES. Across biochemical, histological, molecular, and behavioral measures, RES consistently attenuated MEP-induced damage. Its neuroprotective effects appear to work through multiple pathways—boosting antioxidant defenses, calming inflammatory responses, and blocking apoptotic signaling. These findings suggest that RES holds real promise as a therapeutic agent for mitigating neurotoxic damage from environmental toxicants. Translating these results to human health will require further research, but this study provides a strong foundation for that effort.

Previous studies have demonstrated that oxidative stress signaling pathways play an important role in paraben-induced toxicity and neuronal damage 19. In our study, the concurrent upregulation of *Nrf2* and *ASK1* suggests a coordinated cellular response to oxidative stress: *Nrf2* activation represents a compensatory antioxidant mechanism (the brain's attempt to defend itself), while *ASK1* mediates stress-induced apoptotic signaling (pushing cells toward death). This dual response captures the struggle occurring inside MEP-exposed neurons. Importantly, RES administration significantly reduced the expression levels of these inflammatory mediators, highlighting its strong anti-inflammatory activity in brain tissue. The anti-inflammatory effects of RES are widely documented and attributed to its ability to suppress inflammatory signaling pathways and inhibit cytokine production 11.

## Figures and Tables

**Figure 1:**
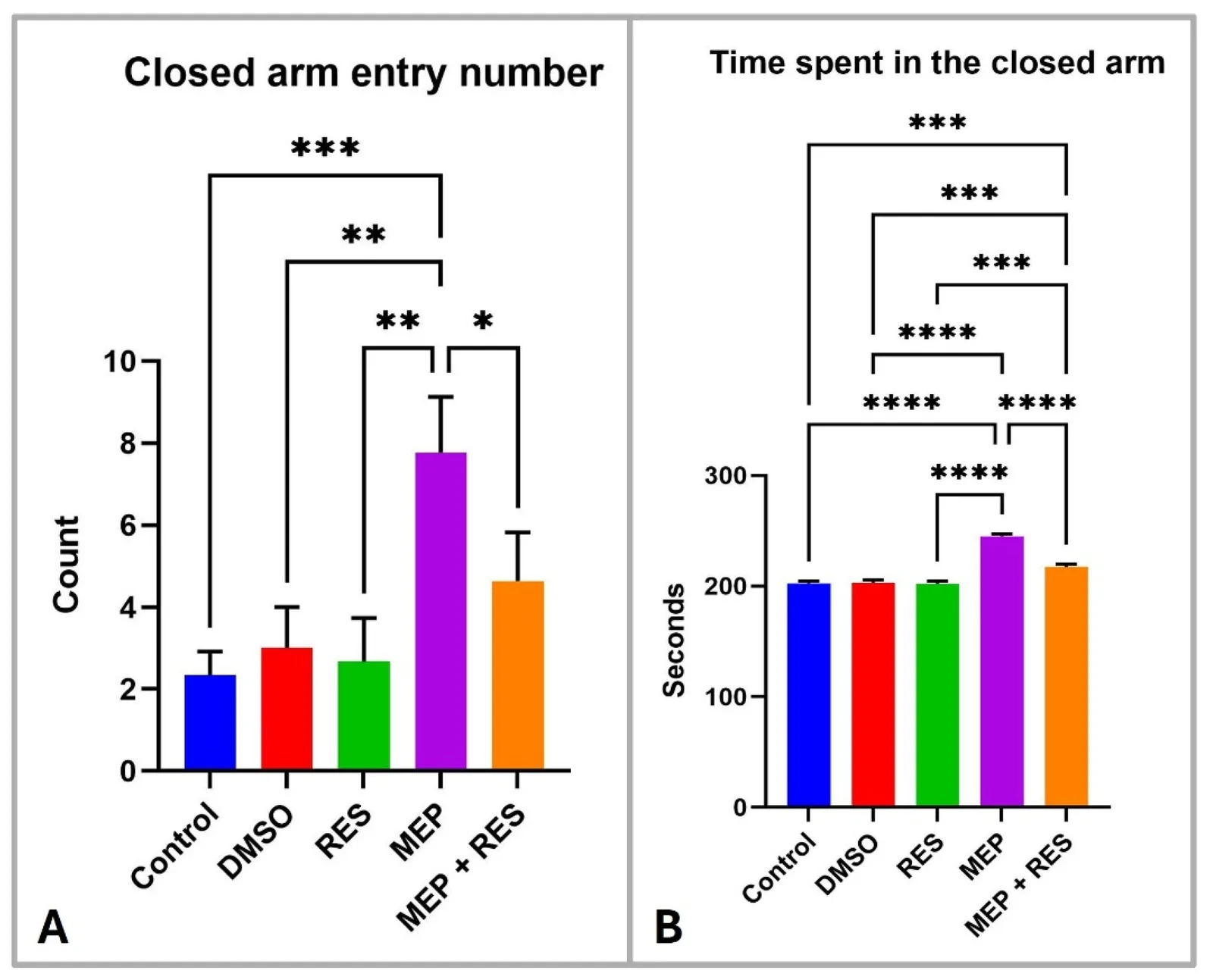
Effects of MEP and RES on anxiety-like behavior in the Elevated Plus Maze (EPM). (A) Closed arm entry number. MEP-treated rats showed a significant increase in closed-arm entries compared with the Control, DMSO, and RES groups, indicating heightened anxiety-like behavior. Co-treatment with RES (MEP + RES) partially reduced this effect. (B) Time spent in the closed arm. MEP markedly increased the duration spent in the closed arm relative to all other groups, while RES co-administration attenuated this increase. Data are presented as mean ± SEM. Statistical analysis was performed using one-way ANOVA followed by Tukey's HSD post-hoc test. Significance levels: *p < 0.05, **p < 0.01, ***p < 0.001, ****p < 0.0001.

**Figure 2:**
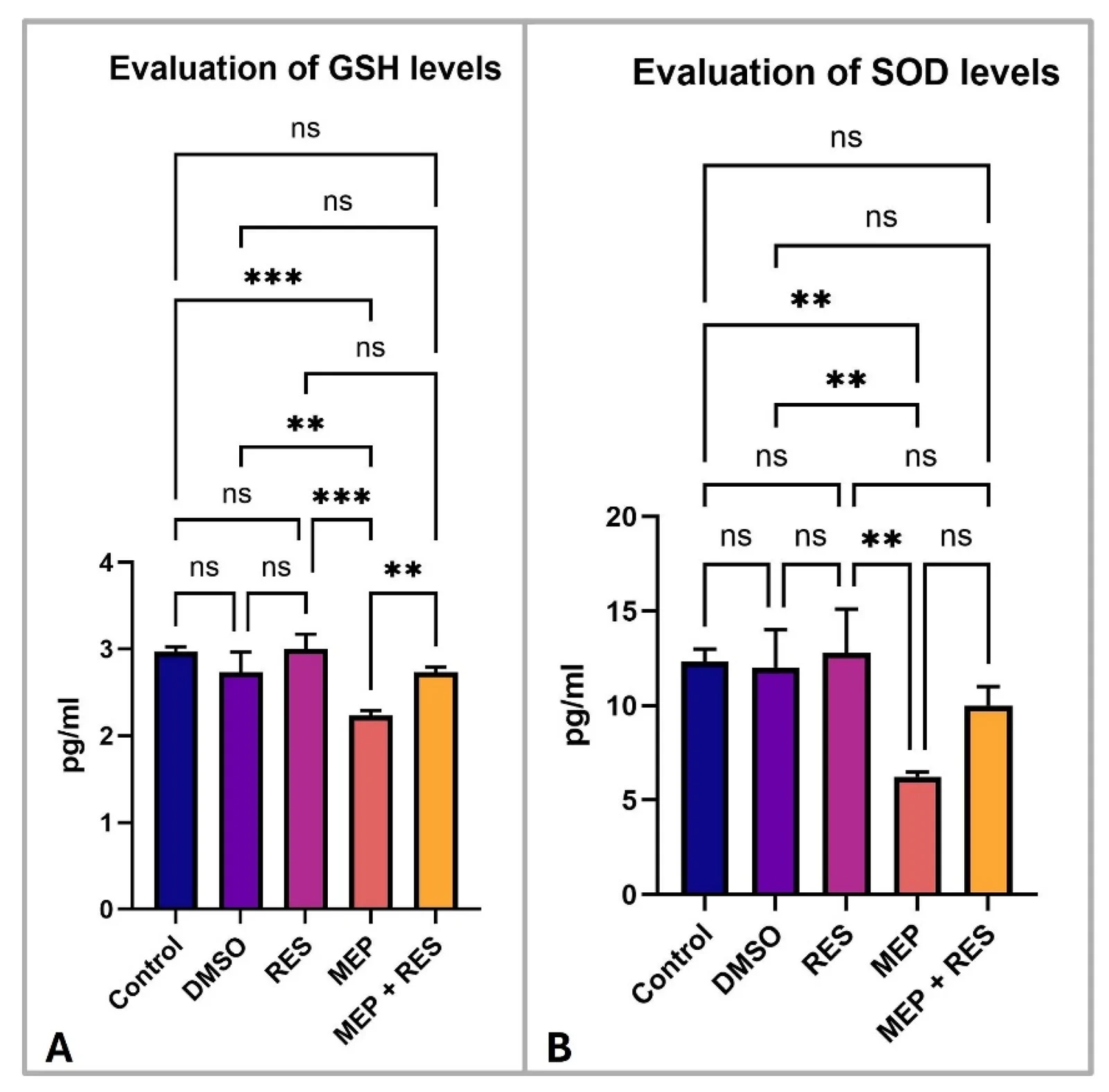
Effects of MEP and RES on antioxidant biomarkers (GSH and SOD) in brain tissue. (A) GSH levels (pg/ml). MEP exposure caused a significant depletion in GSH compared with the control group (*p* < 0.01), while co-treatment with RES markedly restored GSH levels (*p < 0.001 vs. MEP). No significant differences were observed between control, DMSO, and RES groups. (B) SOD levels (pg/ml). MEP significantly reduced SOD activity (*p* < 0.01 vs. control), whereas RES co-administration partially ameliorated this decline** (***p* < 0.01 vs. MEP). Control, DMSO, and RES groups showed no significant differences. Data are presented as mean ± SEM. Statistical comparisons were performed using one-way ANOVA followed by Tukey's HSD post-hoc test.

**Figure 3:**
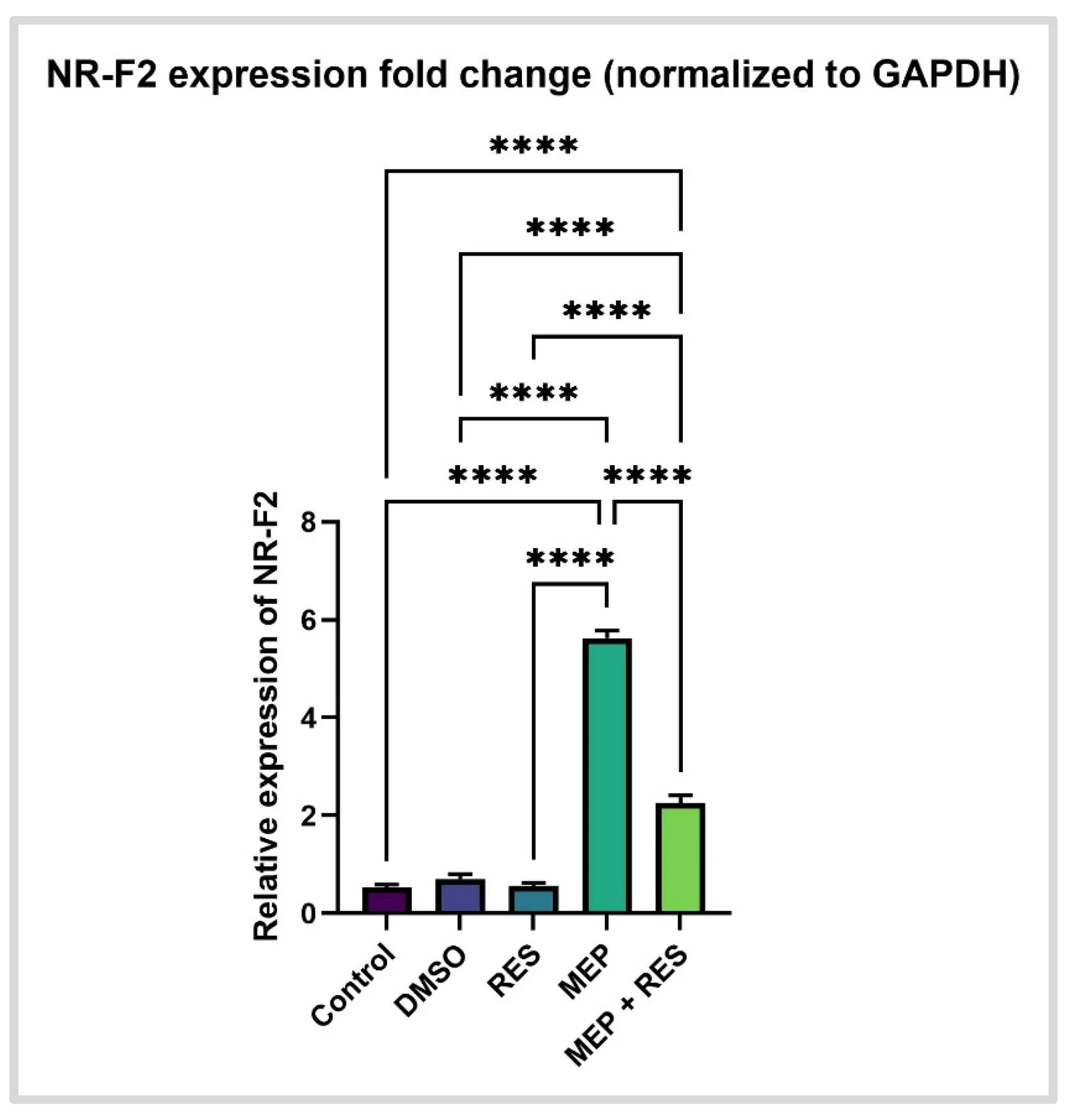
*Nrf2* gene expression in Brain tissue following MEP and RES treatments. Relative mRNA expression of NR-F2, normalized to GAPDH. MEP exposure induced a marked upregulation of NR-F2 expression (****p < 0.0001 vs. Control), indicating strong activation of the oxidative-stress response pathway. Co-treatment with RES significantly reduced this MEP-induced overexpression (****p < 0.0001 vs. MEP), while Control, DMSO, and RES groups showed comparable baseline expression levels. Data are presented as mean ± SEM, and statistical analysis was performed using one-way ANOVA followed by Tukey's HSD post-hoc test.

**Figure 4:**
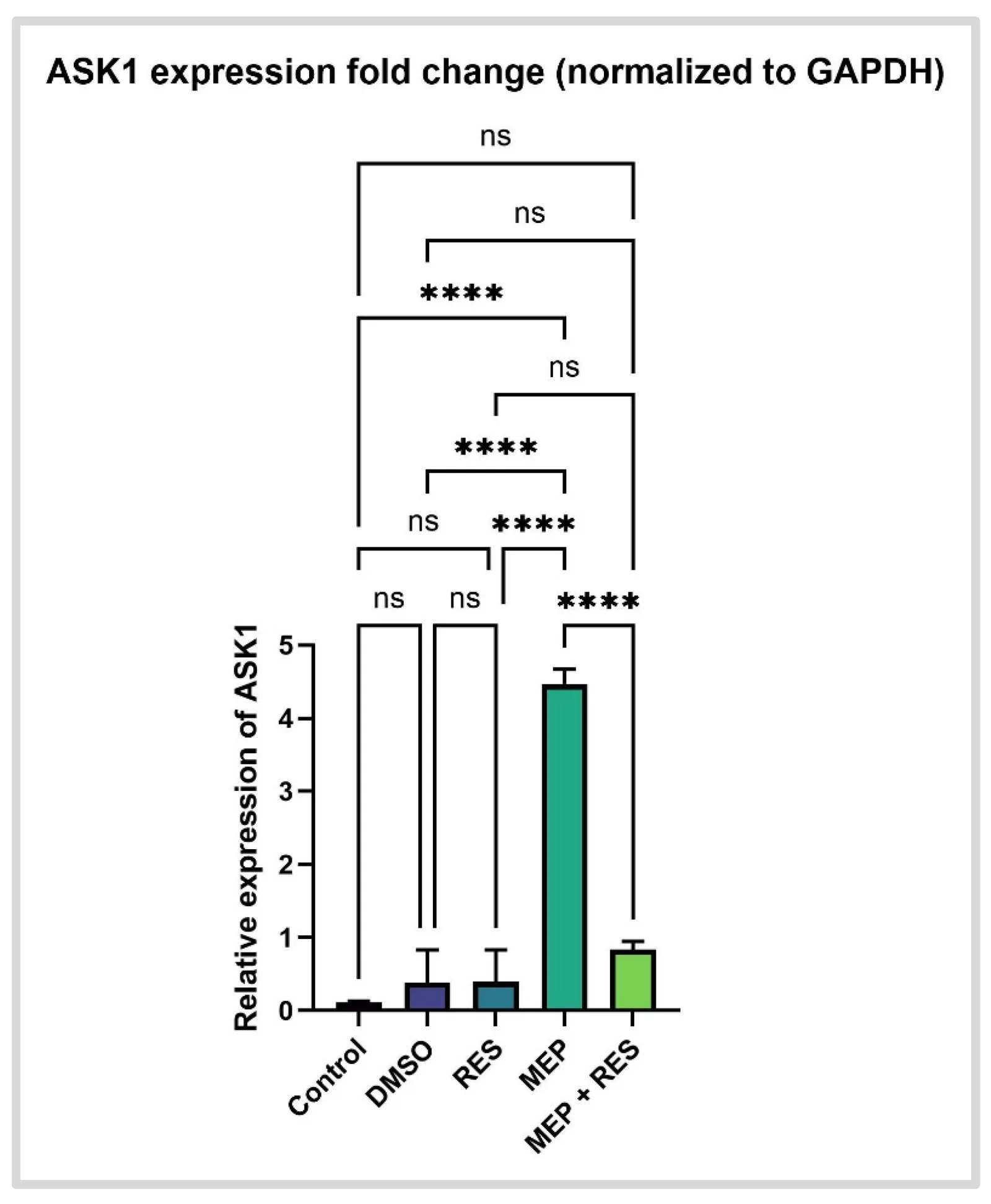
Bar graph showing upregulation of *ASK1* expression in brain. *ASK1* expression remained low in the control, DMSO, and RES groups, whereas MEP exposure induced a marked upregulation of *ASK*1 (*p* < 0.0001). Co-treatment with MEP + RES significantly reduced ASK1 expression compared with MEP alone (*p* < 0.0001), indicating a protective modulatory effect of RES. Data are presented as mean ± SEM. Statistical comparisons were performed using one-way ANOVA followed by Tukey's HSD post-hoc test; ns = not significant.

**Figure 5:**
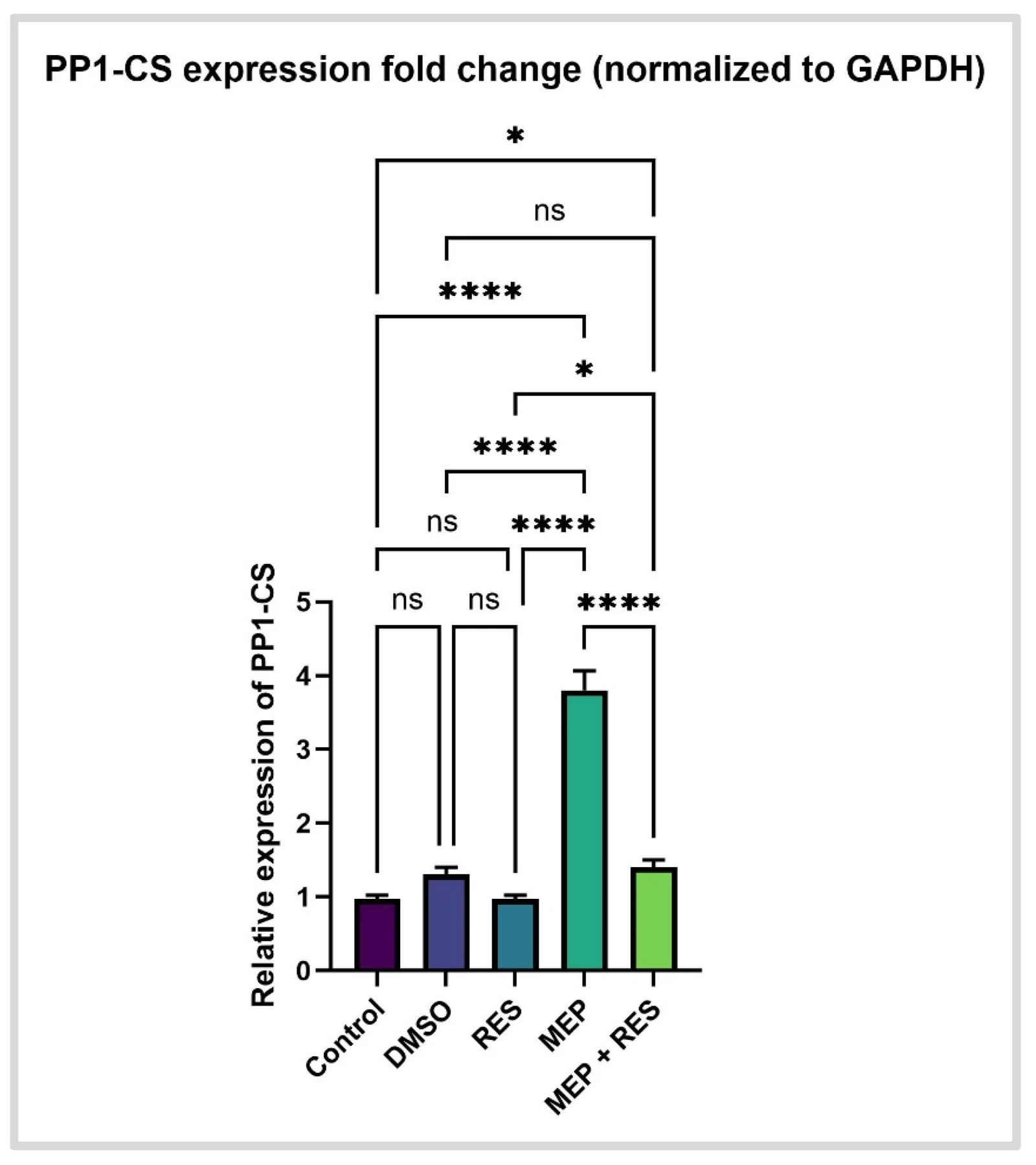
Bar graph showing upregulation of *PP-1cs* expression in brain. *PP-1cs* expression remained low and comparable in the control, DMSO, and RES groups (ns), whereas MEP exposure induced a significant upregulation of PP-1cs (*p* < 0.0001). Co-treatment with MEP + RES markedly reduced *PP-1cs* expression compared with MEP alone (*p < 0.05; ****p < 0.0001), indicating a protective modulatory effect of RES. Data are expressed as mean ± SEM. Statistical analysis was performed using one-way ANOVA followed by a Tukey's HSD post-hoc test.

**Figure 6:**
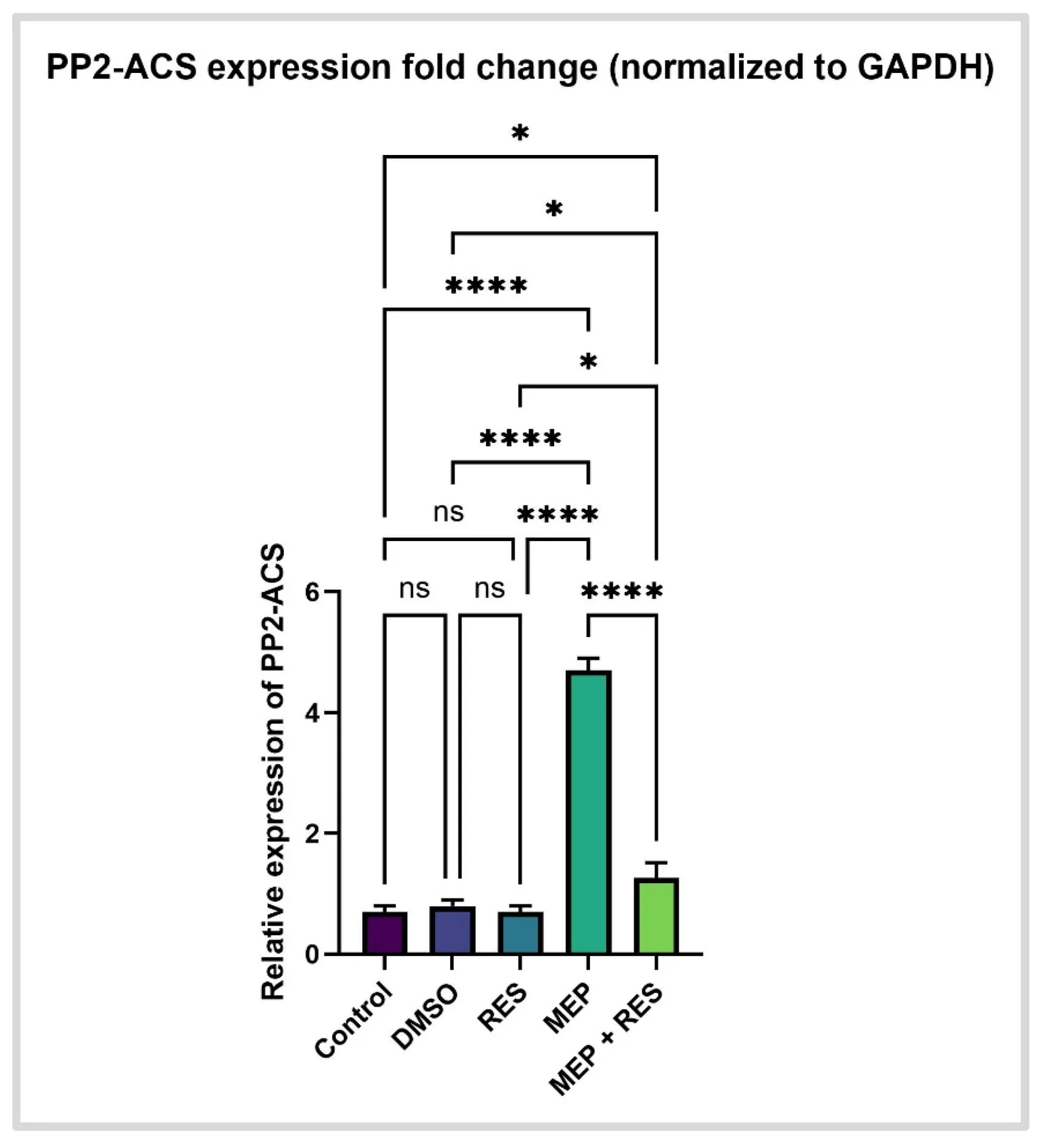
Bar graph showing upregulation of *PP-2Acs* expression in brain. *PP-2Acs* expression remained low and comparable in the control, DMSO, and RES groups (ns). In contrast, MEP exposure induced a marked and highly significant upregulation of *PP-*2Acs (****p < 0.0001). Co-treatment with MEP + RES significantly attenuated this elevation (*p < 0.05; ****p < 0.0001), demonstrating the modulatory protective effect of RES. Data are presented as mean ± SEM. Statistical analysis was performed using one-way ANOVA followed by Tukey's HSD post-hoc test.

**Figure 7:**
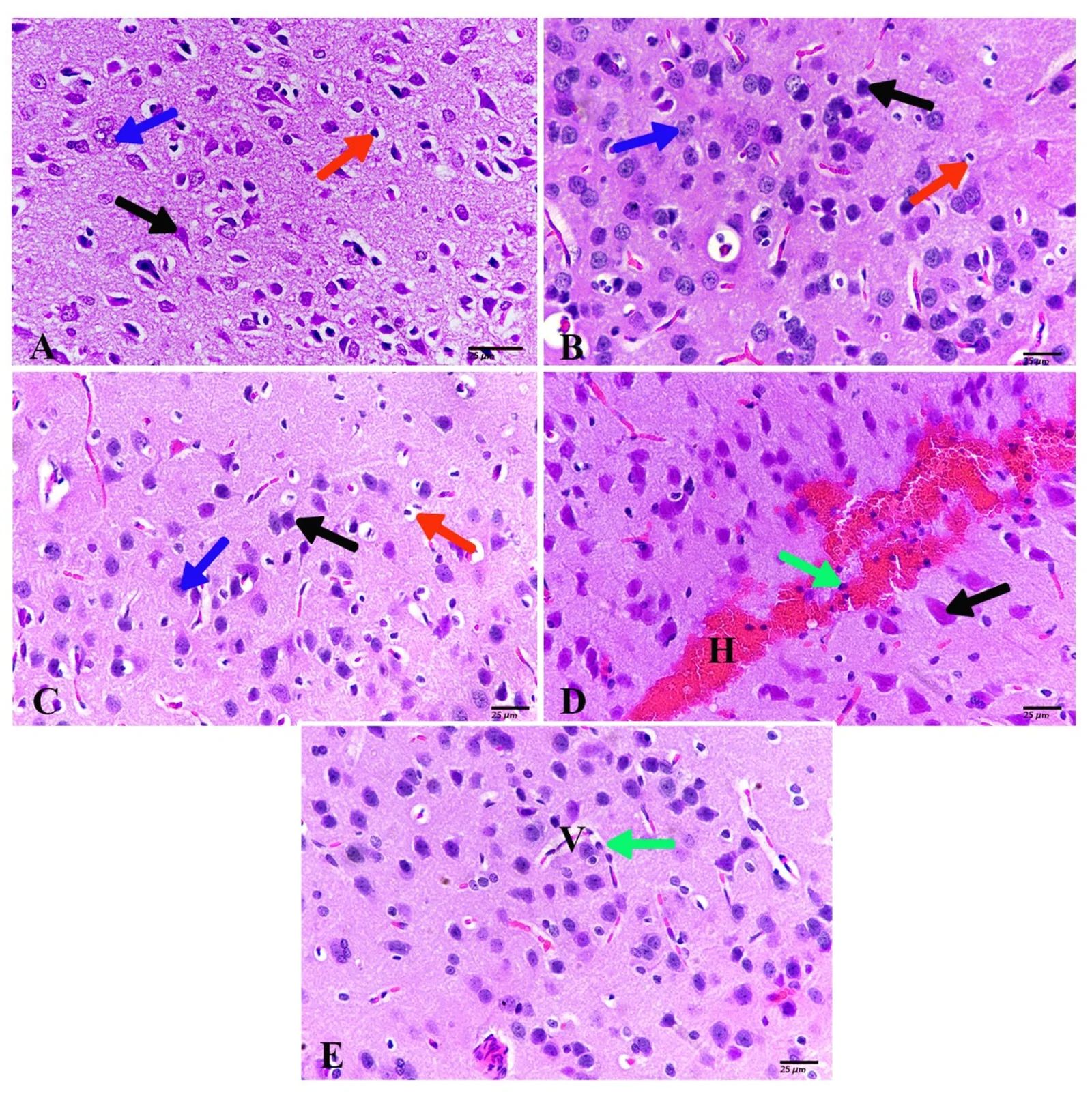
Photomicrographs of the cerebral cortex. (A) Control cerebral cortex showing normal appearance. (B) Cerebral cortex of animals treated with DMSO revealing no pathological changes. (C) Cerebral cortex of animals treated with RES (20 mg/kg) showing healthy cortex. (D) Cerebral cortex of animals treated with MEP (200 mg/kg) exhibiting severe hemorrhage, enlargement of eosinophilic pyramidal neurons, and inflammatory cells. (E) Cerebral cortex of animals treated with MEP + RES displaying a small number of inflammatory cells and dilated blood vessels. (H&E, 400×). Pyramidal neuron (black arrow), granular neuron (blue arrow), glial cell (red arrow), hemorrhage (H), blood vessel (V).

**Figure 8:**
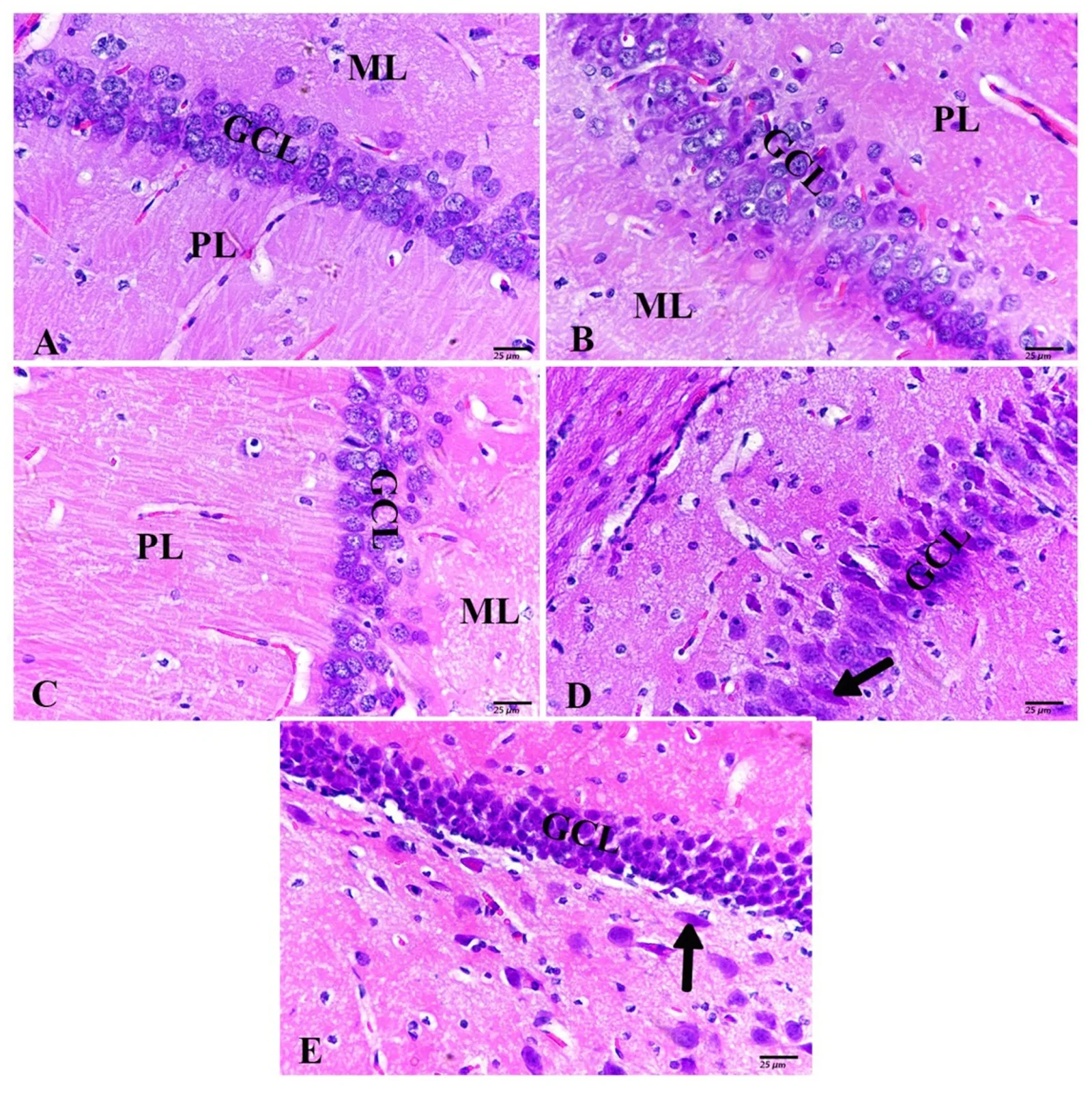
Photomicrographs of the hippocampus. (A) Control hippocampus showing normal structure. (B) Hippocampus of animals treated with DMSO revealing no pathological alterations. (C) Hippocampus of animals treated with RES (20 mg/kg) showing healthy hippocampus. (D) Hippocampus of animals treated with MEP (200 mg/kg) exhibiting enlarged neurons. (E) Hippocampus of animals treated with MEP + RES displaying a small number of distorted neurons. (H&E, 400×). Granular cell layer (GCL), molecular layer (ML), polymorphic layer (PL), enlarged and distorted neurons (black arrow).

**Figure 9:**
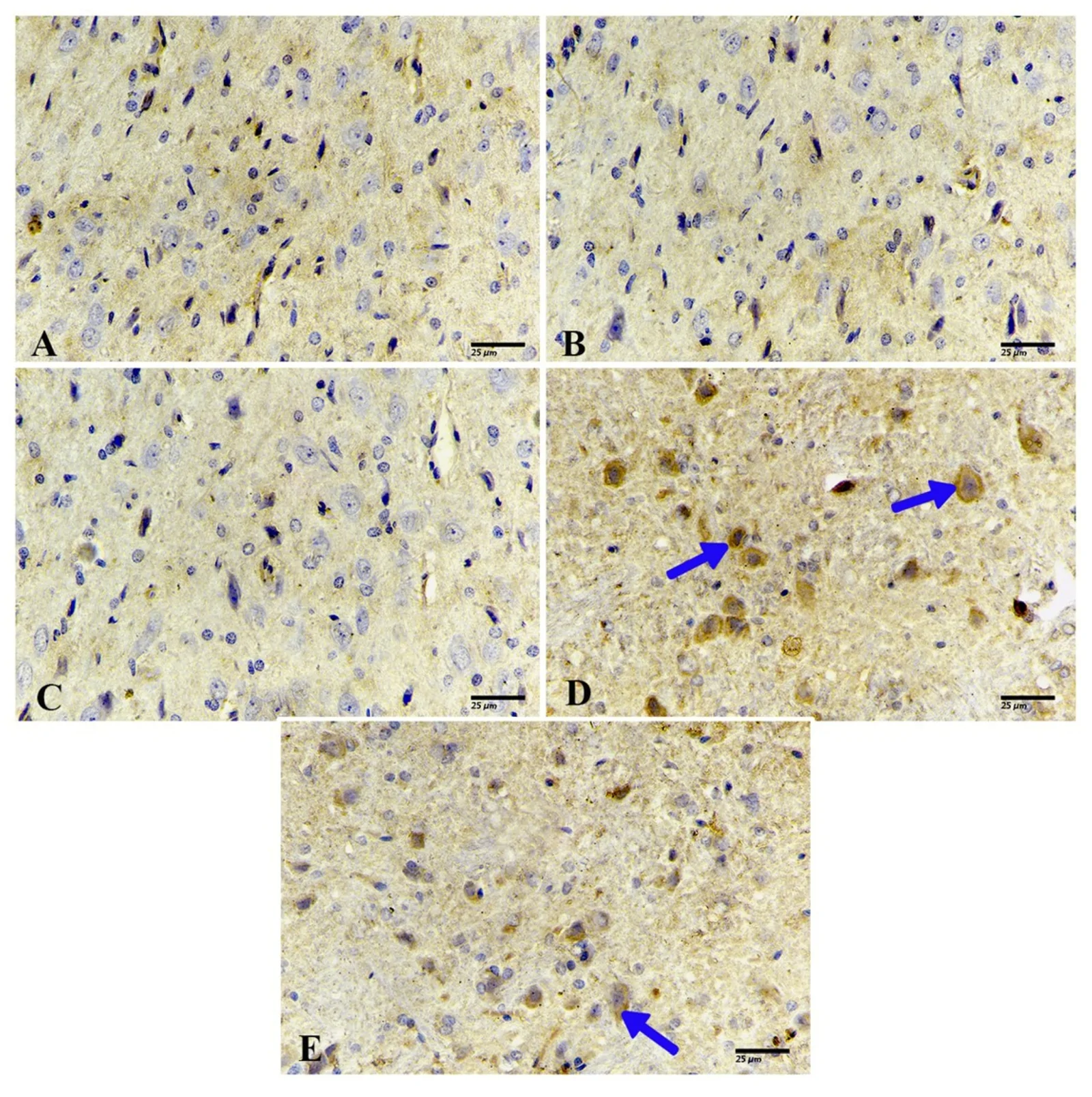
Photomicrographs of the cerebral cortex stained for TNF-α expression. (A) Control cerebral cortex showing weak immunoreactivity. (B) Cerebral cortex of animals treated with DMSO showing weak immunoreactivity. (C) Cerebral cortex of animals treated with RES (20 mg/kg) showing weak immunoreactivity. (D) Cerebral cortex of animals treated with MEP (200 mg/kg) exhibiting intense immunoreactivity. (E) Cerebral cortex of animals treated with MEP + RES displaying moderate immunoreactivity. (ABC, 400×). Expression is indicated by blue arrows.

**Figure 10:**
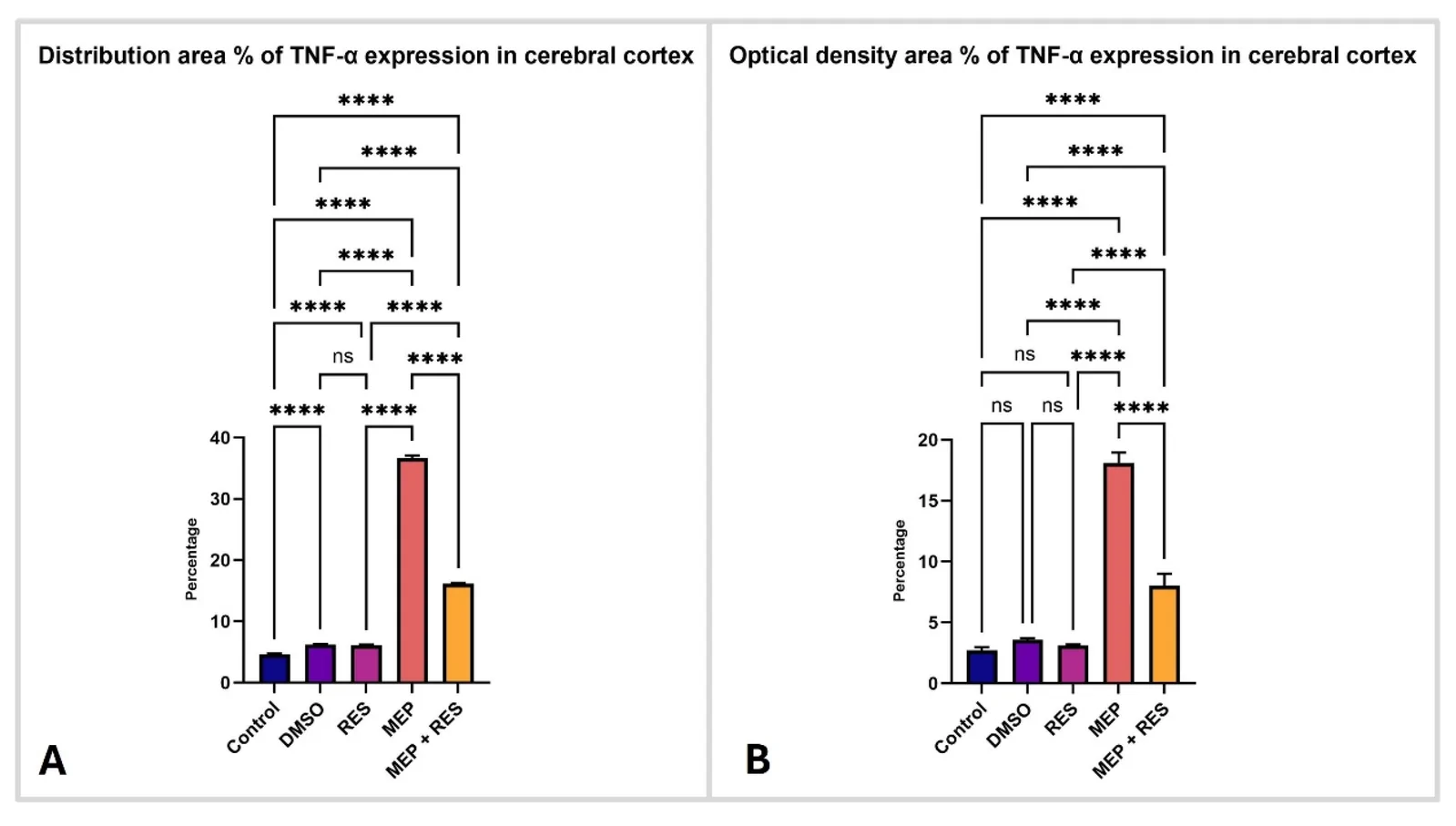
Quantitative analysis of TNF-α immune expression in the cerebral cortex across experimental groups. (A) Distribution area (%) of TNF-α-positive immunoreactivity showing marked elevation in the MEP group compared with control, DMSO, and RES groups (*p* < 0.0001), while co-treatment with MEP + RES significantly reduced TNF-α expression (*p* < 0.0001), indicating a protective effect of RES. (B) Optical density area (%) of TNF-α immunostaining demonstrating a similar pattern, with maximal intensity in MEP-treated animals and partial attenuation in the MEP + RES group. Data are expressed as mean ± SEM. Statistical analysis was performed using one-way ANOVA followed by Tukey's HSD post-hoc test; ns = not significant.

**Figure 11:**
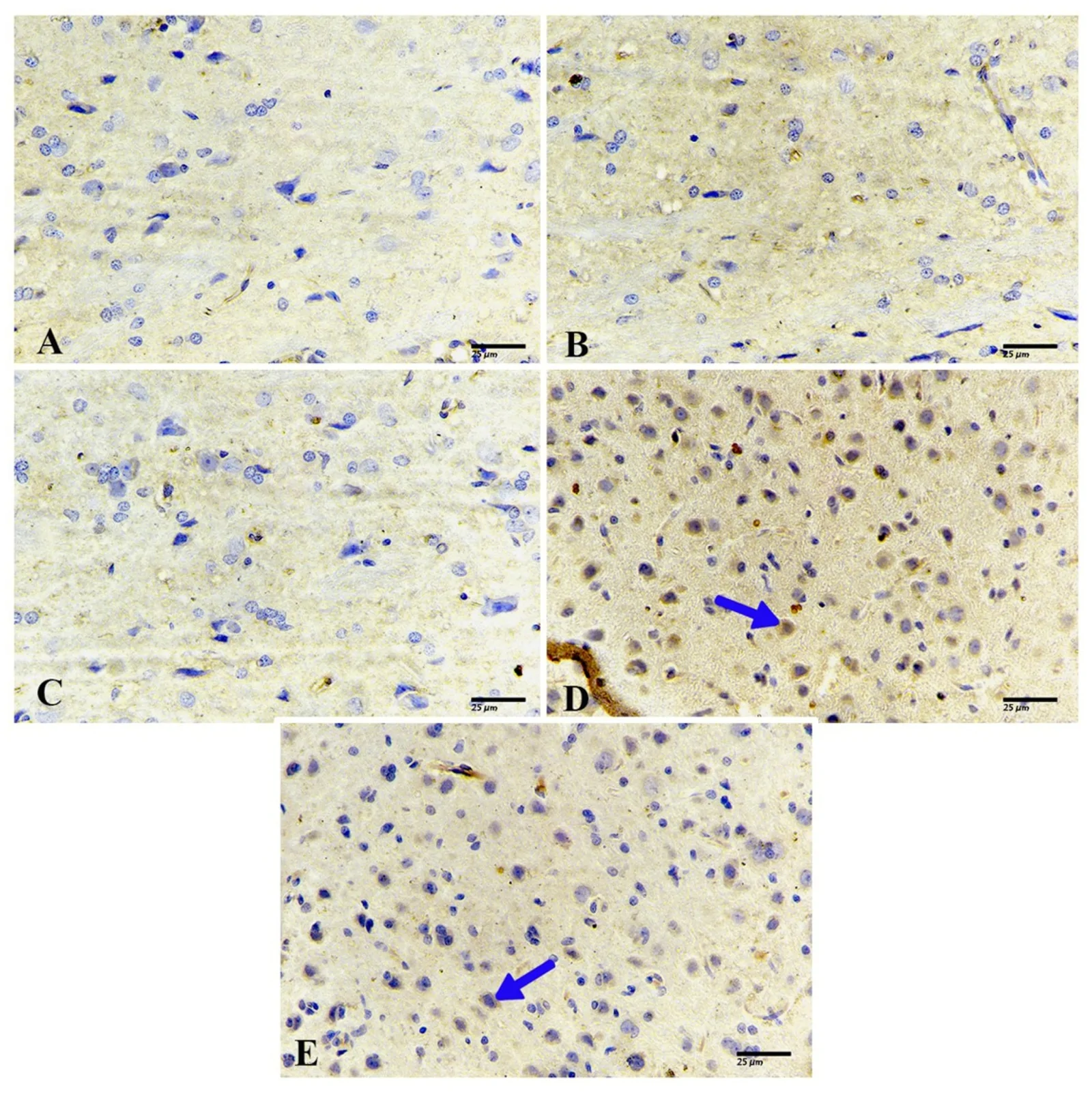
Photomicrographs of the cerebral cortex stained for IL-6 expression. (A) Control cerebral cortex showing weak immunoreactivity. (B) Cerebral cortex of animals treated with DMSO showing weak immunoreactivity. (C) Cerebral cortex of animals treated with RES (20 mg/kg) showing weak immunoreactivity. (D) Cerebral cortex of animals treated with MEP (200 mg/kg) exhibiting intense immunoreactivity. (E) Cerebral cortex of animals treated with MEP + RES displaying moderate immunoreactivity. (ABC, 400×). Expression is indicated by blue arrows.

**Figure 12:**
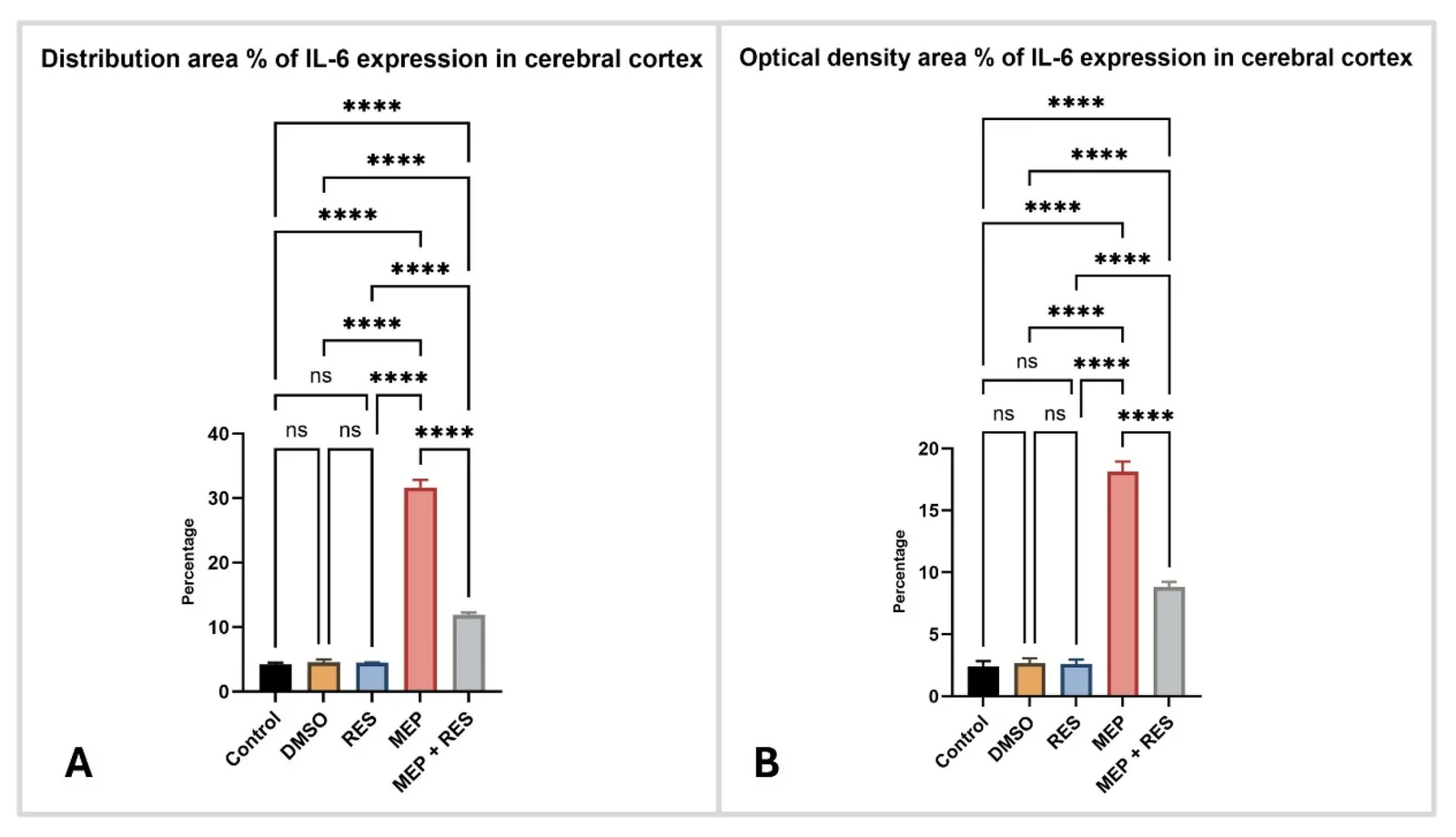
Quantitative analysis of IL-6 immune expression in the cerebral cortex across experimental groups. (A) Distribution area (%) of IL-6-positive immunoreactivity showing a marked elevation in the MEP group compared with control, DMSO, and RES groups (*p* < 0.0001), while co-treatment with MEP + RES significantly reduced IL-6 expression (p < 0.0001), indicating a protective effect of RES. (B) Optical density area (%) of IL-6 immunostaining demonstrating a similar pattern, with maximal intensity in MEP-treated animals and partial attenuation in the MEP + RES group. Data are expressed as mean ± SEM. Statistical analysis was performed using one-way ANOVA followed by Tukey's HSD post-hoc test; ns = not significant.

**Figure 13:**
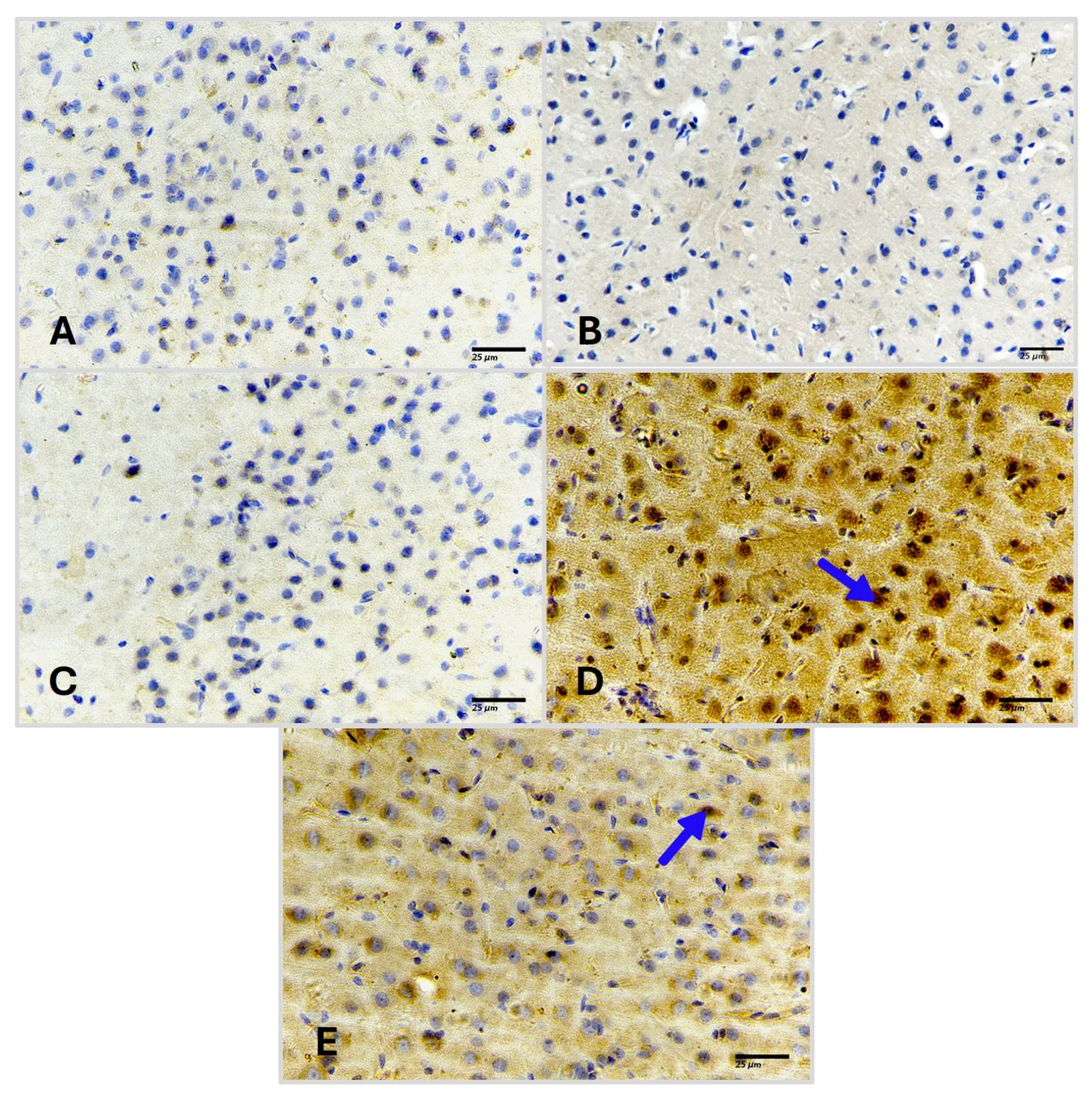
Photomicrographs of the cerebral cortex stained for BAX expression. (A) Control cerebral cortex showing negative immunoreactivity. (B) Cerebral cortex of animals treated with DMSO showing negative immunoreactivity. (C) Cerebral cortex of animals treated with RES (20 mg/kg) showing negative immunoreactivity. (D) Cerebral cortex of animals treated with MEP (200 mg/kg) exhibiting intense immunoreactivity. (E) Cerebral cortex of animals treated with MEP + RES showing reduced immunoreactivity. (ABC, 400×). Expression is indicated by blue arrows.

**Figure 14:**
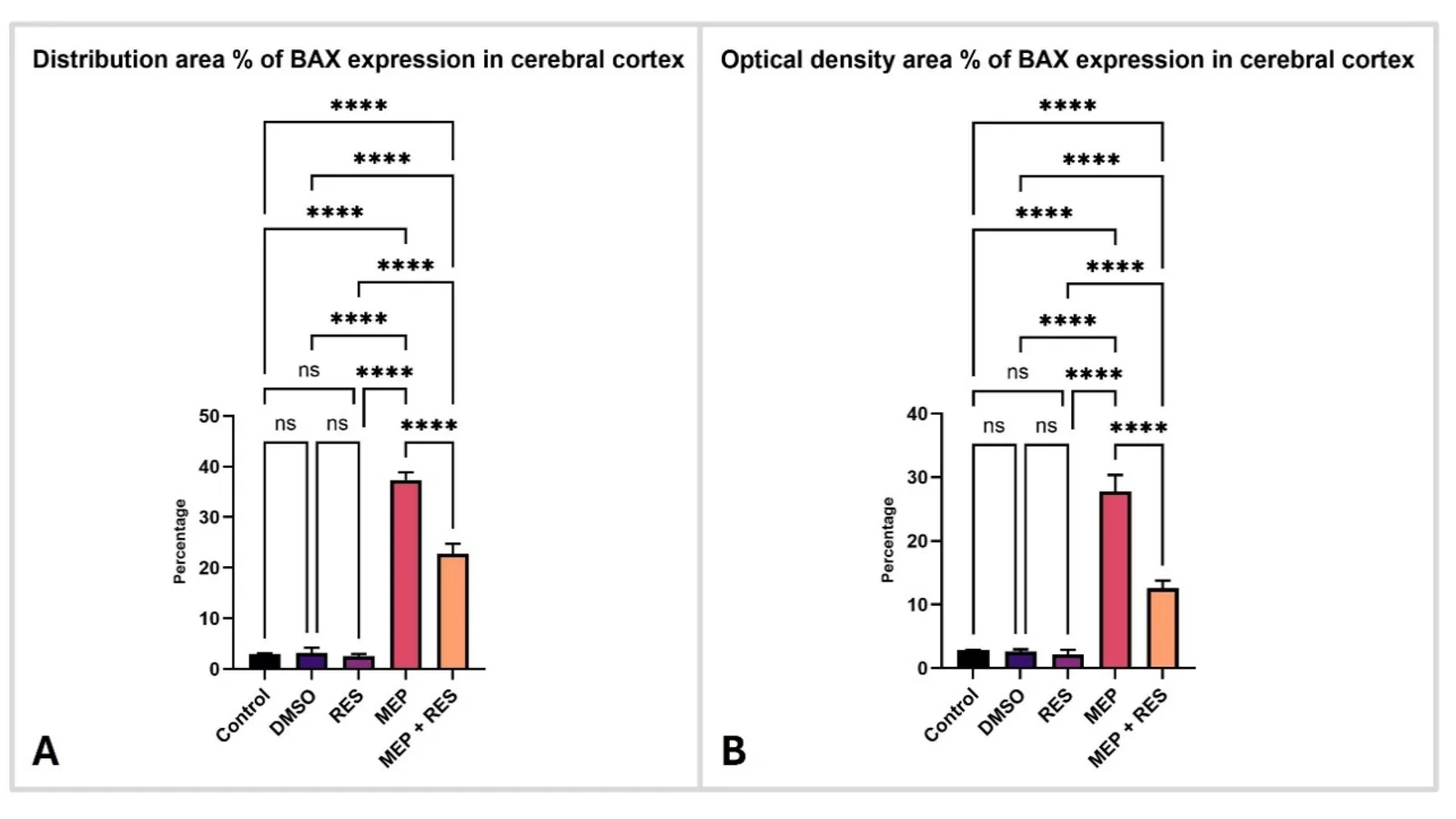
Quantitative analysis of BAX immune expression in the cerebral cortex across experimental groups. (A) Distribution area (%) of BAX-positive immunoreactivity showing a marked and highly significant elevation in the MEP group compared with control, DMSO, and RES groups (*p* < 0.0001). Co-treatment with MEP + RES significantly reduced BAX expression relative to MEP alone (*p* < 0.0001), indicating a protective anti-apoptotic effect of RES. (B) Optical density area (%) of BAX immunostaining demonstrating the same pattern, with maximal staining intensity in the MEP group and partial attenuation in the MEP + RES group. Data are expressed as mean ± SEM. Statistical analysis was performed using one-way ANOVA followed by appropriate post-hoc testing; ns = not significant.

**Figure 15:**
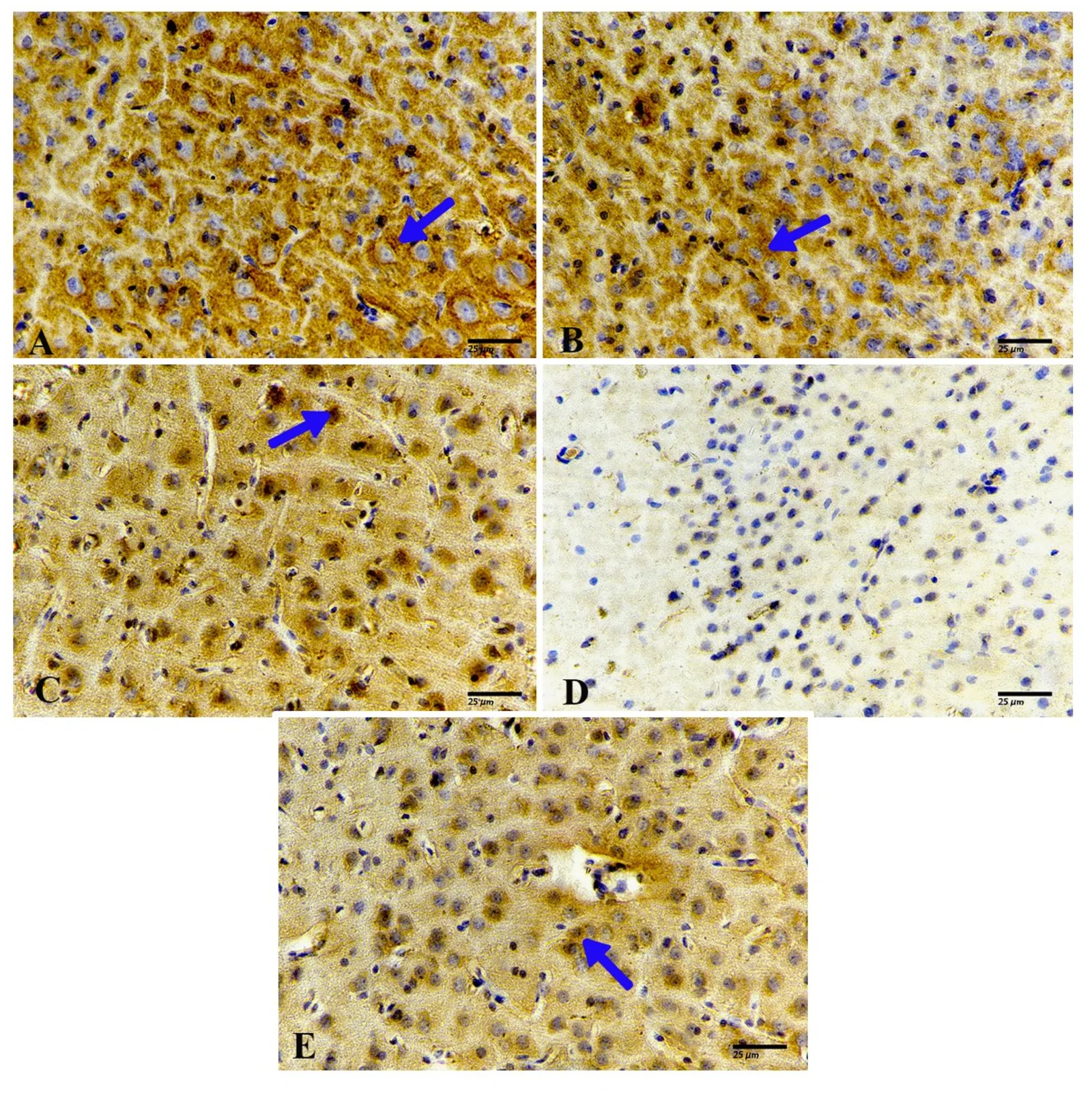
Photomicrographs of the cerebral cortex stained for BCL-2 expression in female rats following methylparaben (MEP) and resveratrol (RES) treatments (ABC method, ×400 magnification). (A) Control cerebral cortex showing intense expression. (B) DMSO-treated cerebral cortex showing strong expression. (C) RES-treated cerebral cortex (20 mg/kg) showing intense expression. (D) MEP-treated cerebral cortex (200 mg/kg) displaying weak expression. (E) MEP + RES-treated cerebral cortex showing moderate immune expression. Expression is indicated by blue arrows.

**Figure 16:**
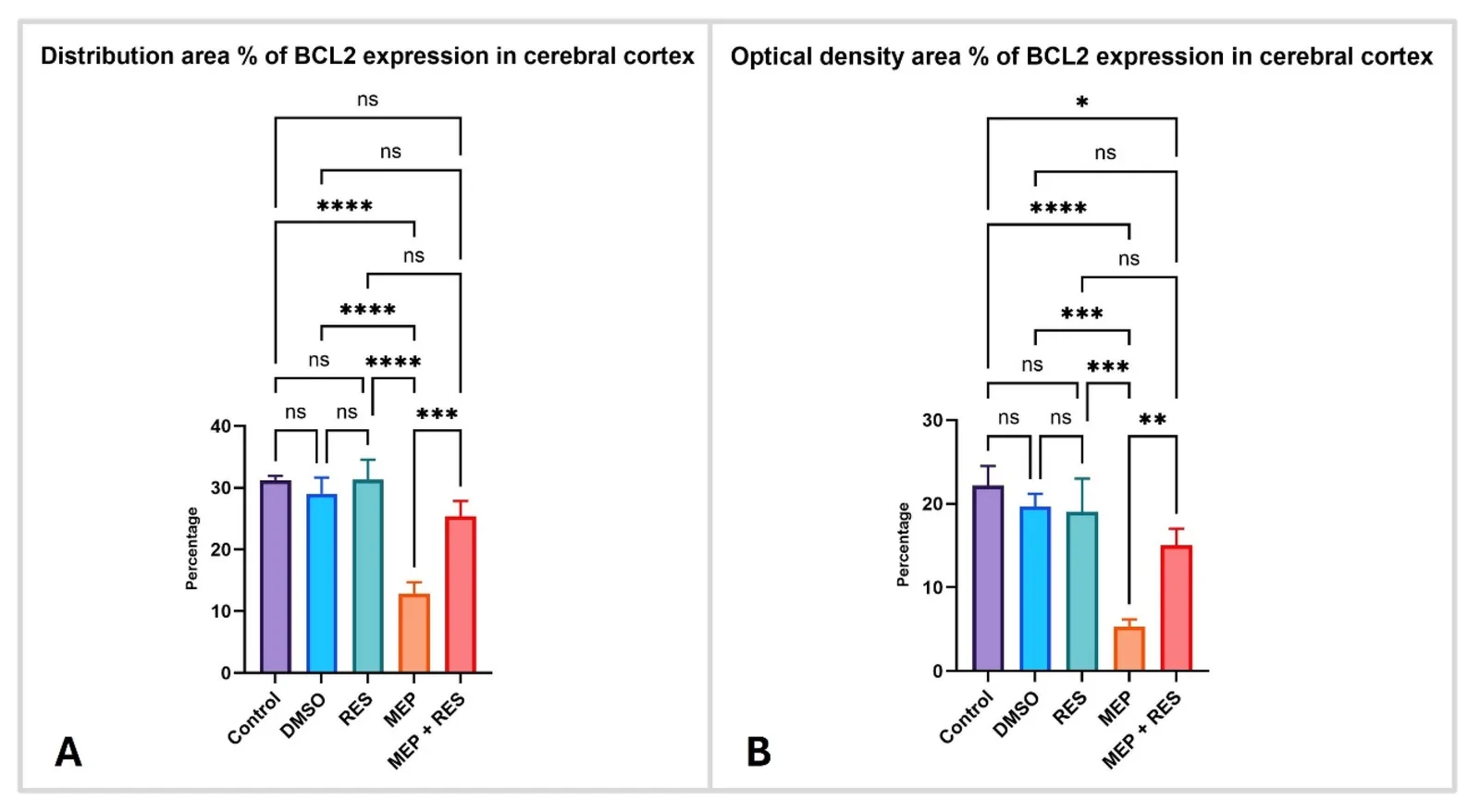
Quantitative analysis of BCL-2 immune expression in the cerebral cortex of female rats across experimental groups. (A) Distribution area (%) of BCL-2-positive immunoreactivity showing significantly reduced expression in the MEP group compared with Control, DMSO, and RES groups (***p* < 0.001; ****p* < 0.0001). Co-treatment with MEP + RES partially restored BCL-2 expression relative to MEP alone (*p* < 0.05; **p* < 0.01; ***p* < 0.001), indicating an anti-apoptotic protective effect of RES. (B) Optical density area (%) of BCL-2 immunostaining demonstrating a similar pattern, with marked suppression in the MEP group and partial recovery in the MEP + RES group. Data are presented as mean ± SEM (n = 10 per group). Statistical analysis was performed using one-way ANOVA followed by appropriate post-hoc testing; ns = not significant.

**Table 1:** Primer sequences used for RT-qPCR analysis

Gene name	Forward primer	Reverse primer	Access NO.
*NRF2*	5′ CACATCCAGACAGACACCAGT-3′	5′ CTACAAATGGGAATGTCTCTGC-3′	NM_031789
*GAPDH*	5′CAGGCATATGGTGGTCCATAGAG-3′	5′ TCATGGGATCCACCTGCAGC-3′	NM_017008
PP-1cs	5′CTGGATTCCATCATCGGGCG3′	5′AGGCAAAGACCACGGATCTC3′	NM_031527
PP-2Acs	5′GTACTGCACAAGTCACCGCA3′	5′CCAGTCGTGCCCACTGATAC3′	NM_017039.3
Ask1	5′TATGTGGTTGCACGGAGAGG3′	5′AGAAACGGAGCCATACTCGC3′	NM_001277694.1

## Data Availability

Available upon request**.**
